# Theta burst stimulation for upper limb motor dysfunction in patients with stroke: A protocol of systematic review and meta-analysis

**DOI:** 10.1097/MD.0000000000017929

**Published:** 2019-11-15

**Authors:** Xiao-bo Liu, Jian-guo Zhong, Xi-li Xiao, Yu-xi Li, Yi-jie Huang, Yong-guo Liu, Chi Zhang, Rong-jiang Jin, Tian-yu Liu

**Affiliations:** a School of Health Preservation and Rehabilitation, Chengdu University of Traditional Chinese Medicine; b Department of Rehabilitation, Second Affiliated Hospital of Chengdu Medical College (China National Nuclear Corporation 416 Hospital); c Hospital of Chengdu University of Traditional Chinese Medicine; d School of Acupuncture-Moxibustion and Tuina, The Third Affiliated Hospital, Chengdu University of Traditional Chinese Medicine; e Knowledge and Data Engineering Laboratory of Chinese Medicine, School of Information and Software Engineering, University of Electronic Science and Technology of China; f School of Sport, Chengdu University of Traditional Chinese Medicine, Chengdu, Sichuan, China.

**Keywords:** meta-analysis, randomized controlled trial, stroke, systematic review, theta burst stimulation, transcranial magnetic stimulation, upper limb motor dysfunction

## Abstract

**Background:**

Upper limb dysfunction is one of common sequelae of stroke which limits daily activities and decreases quality of life of patients, as well as increasing caregiving burden on families. Theta burst stimulation (TBS) is considered to be a beneficial therapy for post-stroke patients with upper limb motor dysfunction, but there is a lack of a high quality evidence. We aim to investigate the effectiveness and safety of TBS for upper limb motor dysfunction in patients with stroke.

**Methods:**

The following databases will be searched: PubMed, EMBASE, The Cochrane Library, Web of Science, China Biology Medicine (CBM), China National Knowledge infrastructure (CNKI), Technology Periodical Database (VIP) and WanFang Data from the inception to October 2019. All relevant randomized controlled trials (RCTs) using TBS to treat poststroke patients with upper limb motor dysfunction will be included. The primary outcome is Upper Limb Fugl-Meyer Assessment (UL-FMA). Secondary outcomes will include Action Research Arm Test (ARAT), Box and Block Test (BBT), Wolf Motor Function Test (WMFT), Motor Assessment Scale (MAS), Nine Hole Peg Test (NHPT), Grip strength and other scales evaluating the upper limb motor function. Adverse effects will also be evaluated. Two reviewers will screen studies, extract data and assess the risk of bias of included studies independently. Data analysis will be conducted using Review Manager software (RevMan, version 5.3.5) and R software (version 3.6.1).

**Results:**

Our SR will be conducted according to AMSTAR 2.0 and reported in compliance with PRISMA. The findings of this SR will be disseminated through peer-reviewed publications or conference presentations.

**Conclusion:**

Our study will provide evidence for the effectiveness and safety of theta burst stimulation for upper limb motor dysfunction in patients with stroke.

**Ethics and dissemination:**

This systematic review (SR) does not require formal ethical approval since no privacy health information will be included. The findings of this SR will be disseminated through peer-reviewed publications or conference presentations.

**PROSPERO registration number:**

CRD42019142462.

## Abstract 2

Supplemental Digital Content is available in the text

## 1 Introduction

Stroke is one of the most devastating neurological conditions and the leading cause of long-term disability.^[1,2]^ About 85% stroke survivors experience hemiparesis, resulting in 55% to 75% survivors suffer from upper limb functional limitation.^[3]^ Limited upper limb function will cause difficulty in daily activities such as eating, dressing, and personal care, which is associated with low quality of life,^[4,5]^ as well as increasing caregiving burden on families.^[6]^ Meanwhile, a Swedish study found that the total average cost ranged from €21,000 to €120,000 created by first year of poststroke care which brings extensive economic burden to families and society, and more severe motor functional disability means higher costs.^[7]^ Therefore, to improve upper limb dysfunction is crucial for rehabilitation after stroke.^[8]^

Noninvasive brain stimulation is considered to be an effective complementary therapy for poststroke rehabilitation,^[9]^ of which, repetitive transcranial magnetic stimulation (rTMS) has been widely used in the rehabilitation of motor, speech, and cognitive dysfunction after stroke.^[10–12]^ Theta burst stimulation (TBS) is a novel form of rTMS, which can improve brain plasticity by generating a facilitatory or inhibitory effects on synaptic transmission.^[13]^ Intermittent and continuous theta burst stimulation (iTBS, cTBS) are 2 major patterns and the most commonly used methods in TBS. Based on interhemispheric competition model, the balance between hemisphere could be disrupted after stroke,^[14,15]^ causing inhibition from unaffected hemisphere (UH) to affected hemisphere (AH) to increase and inhibition from AH to UH to decrease,^[14,16]^ which further influence the recovery of upper limb function.^[15,17]^ iTBS and cTBS can increase AH excitability and decrease UH excitability, respectively.^[13]^ Therefore, the modulation of excitability of hemispheres induced by TBS may help to improve motor dysfunction of poststroke patients.

In recent years, relevant studies have been carried out to observe the effects of TBS on upper limb motor dysfunction after stroke, but there are controversies among these results.^[18–21]^ Three previous systematic reviews (SRs) involved meta-analysis of effects of TBS for poststroke patients with upper limb dysfunction have been conducted, all of the results showed positive.^[22–24]^ Nevertheless, 2 of them did descriptive analysis on the effectiveness of TBS due to inadequate data.^[22,23]^ However, the methodological quality of the 3 SRs were critically low when assessed with the A Measurement Tool to Assess systematic Reviews (AMSTAR 2.0).^[25]^ Besides, lots of trials have been carried out in recent years which are not involved in previous SRs,^[18,21,26–28]^ we plan to conduct a SR and meta-analysis to evaluate the effectiveness and safety of TBS on upper limb motor dysfunction in patients with stroke according to AMSTAR 2.0 and will be reported in compliance with the Preferred Reporting Items for Systematic Reviews and Meta-Analyses (PRISMA).^[25,29]^

## 2 Methods and analysis

### 2.1 Study registration

This study has been registered on PROSPERO (CRD42019142462). This protocol is reported in compliance with the Preferred Reporting Items for Systematic Reviews and Meta-Analyses Protocols (PRISMA-P) statement guidelines.^[30]^ Our SR will be conducted according to AMSTAR 2.0 and reported in compliance with PRISMA.^[25,29]^

### 2.2 Inclusion criteria

#### 2.2.1 Type of studies

We will only include randomized controlled trials (RCTs) published in English and Chinese using TBS as intervention for upper limb motor dysfunction in poststroke patients. There will be no restrictions on publication status.

#### 2.2.2 Type of participants

We will include adult patients (≥18 years old) who were diagnosed with stroke by computed tomography (CT) or magnetic resonance imaging (MRI) and were accompanied by upper limb motor dysfunction (Brunnstrom upper limb and hand stage <VI). There will be no restrictions on gender, race, nation, or disease course.

#### 2.2.3 Type of interventions

RCTs that used TBS therapy for patients with upper limb motor dysfunction after stroke will be included.

#### 2.2.4 Type of comparators

The comparative intervention could be sham TBS, or no intervention.

### 2.3 Outcome measurements

The primary outcome will be the Upper Limb Fugl-Meyer Assessment. Secondary outcomes will include Action Research Arm Test, Box and Block Test, Wolf Motor Function Test, Motor Assessment Scale, Nine Hole Peg Test, Grip strength, and other scales evaluating the upper limb motor function and adverse effects. Adverse effects are defined as epilepsy, headache, vertigo, and paraesthesia.

### 2.4 Exclusion criteria

We will exclude studies fulfill at least one of the following characteristics: published repeatedly; data cannot be extracted; and full text cannot be obtained through various approaches.

### 2.5 Database and search

Two reviewers (JGZ and XLX) will work on study search. The following databases will be searched: PubMed, EMBASE, The Cochrane Library, Web of Science, China Biology Medicine (CBM), China National Knowledge infrastructure (CNKI), Technology Periodical Database (VIP), and WanFang Database from the inception to October 2019. The keywords such as transcranial magnetic stimulation, TBS, stroke, and RCT will be used for searching. In order to maximize search and collection of relevant articles, we will conduct manual search through reviewing references list of identified studies, relevant reviews, and meta-analysis. Gray literature will also be searched. We have developed the PubMed search strategy (see Appendix 1) and will apply similar strategies in other electronic databases.

### 2.6 Studies selection

After removing duplicate literatures, 2 reviewers (XBL and YXL) will independently screen the titles and abstracts for potentially eligibility studies, then the full texts will be obtained. After that, 2 reviewers will read the full texts to identify candidates according to the predefined inclusion criteria. Authors will be contacted in case of unclear information or missing data. Disagreements will be resolved by discussion or consulting experienced reviewer (JRJ). We will provide a list of the excluded studies and reasons for exclusions. Details of the entire selection procedure will be shown in a PRISMA flow diagram.^[29]^

### 2.7 Data extraction

Data will be extracted from each study by 2 reviewers (YXL and YJH) independently with a predesigned data extraction form, including characteristic information following as: articles (first author, publication year, and country), participant (sample size, age, gender, time since stroke, type of stroke, arm affected by stroke, and degree of upper limb impairment), intervention (interventions treatment, comparisons treatment, stimulation parameters, and duration of treatment), outcome measurements (primary and secondary outcomes), design (study design, number of withdraw, and duration of follow-up), and sources of funding. If the data were not reported in original article, we will find them from ClinicalTrails (www.clinicaltrials.gov) or contact the original authors. If the included RCTs involve multiple groups, only groups consistent with the objectives of this SR will be extracted. Discrepancy will be resolved through team discussion.

### 2.8 Assessment of risk of bias

Risk of bias will be assessed independently by 2 reviewers (CZ and YGL) using the Cochrane Collaboration risk of bias tool including the following items: random sequence generation, allocation concealment, blind subjects and therapists, blind assessors, incomplete outcome data, selective outcome reporting, and other bias. We will assessed the risk of bias of each included studies as low, unclear, and high risk of bias according to an approach provided by Cochrane Handbook for Systematic Reviews of Interventions. In case of disagreements, a 3rd reviewer (TYL) will be involved.

### 2.9 Dealing with missing data

The authors will be contacted in case of unclear information and missing data. If there is no reply, we will only analyze the available data. The potential impact of these missing data on the results of this SR will be explained in the discussion section.

### 2.10 Statistical analysis

#### 2.10.1 Data analysis

Meta-analysis will be conducted using Review Manager software (RevMan, version5.3.5) and R software (version 3.6.1). Relative risk (RR) will be used to analyze dichotomous outcomes. For continuous outcomes with the same unit, we will use mean difference (MD) to analyze, and standardized mean difference (SMD) will be used in case of different units. The uncertainty will be expressed with 95% confidence intervals (95% CI), and *P* value <.05 is considered to be statistically significant. We will use *χ*^2^ test to assess the heterogeneity and the *I*^2^ statistic to quantify inconsistency. Fixed-effects model (FEM) will be used if acceptable heterogeneity is found (*I*^2^ < 50%). Random-effect model (REM) will be used where significant statistical heterogeneity exists (*I*^2^ ≥ 50%). If meta-analysis is not available, results will be described qualitatively in the text.

#### 2.10.2 Subgroup analysis

We plan to carry out subgroup analysis if sufficient comparable studies are identified. We will perform subgroup analyses according to the type of TBS (iTBS, cTBS, or iTBS combined with cTBS); time since stroke (≥6 months and <6 months) and type of stroke (ischemic and hemorrhagic stroke). If data is adequate, we will conduct subgroup analyses based on length of follow-up (at least 6 months and <6 months).

#### 2.10.3 Sensitivity analysis

We plan to conduct sensitivity analysis by excluding trails rated as high risk of bias and using random effect models to determine the robustness of the result.

#### 2.10.4 Publication bias

Potential publication bias will be assessed by Funnel plot qualitatively if there are adequate studies. Meanwhile, egger's test will be used to assess potential publication bias quantitatively.

### 2.11 Grading of Recommendations Assessment Development and Evaluation (GRADE)

We will assess the quality of evidence of each outcome with the GRADE system.^[31]^ The quality of the index will be assessed from the following 5 aspects: limitations, inconsistency, indirectness, imprecision, and publication bias. Each outcome will be graded as “high,” “moderate,” “low,” or “very low” in accordance with the GRADE rating standards.

### 2.12 Ethics and dissemination

This SR does not require formal ethical approval since no privacy health information will be included. Results will provide a general overview and evidence concerning the effectiveness and safety of TBS on upper limb motor dysfunction in patients with stroke. The findings of this SR will be disseminated through peer-reviewed publications or conference presentations.

## 3 Discussion

TBS, a novel protocol of rTMS, has attracted extensive attention in recent years. Compared with conventional rTMS, short duration and low intensity stimulus pulses are the advantages of TBS.^[13]^ Applying TBS to stimulate the cerebral cortex, iTBS will produce a long-term potentiation like effect and keep an excitatory effect on cortex.^[32]^ In contrast, cTBS can produce a long-term depression like effect and develop an inhibitory effect.^[32]^ Based on these mechanisms and effects of stimulation, TBS has been used to treat stroke, depression, multiple sclerosis, spinal cord injury, and other neurological and psychiatric diseases.^[33–36]^ In the domains of stroke rehabilitation, TBS has some benefits, such as improving motor, cognitive, and speech dysfunction.^[37–39]^ In the trials which used TBS to improve upper limb motor dysfunction in patients with stroke, some positive results have been observed^[18,19–21]^; however, some RCTs demonstrated negative effects of TBS.^[20,26]^ Therefore, whether TBS is effective for poststroke upper limb motor dysfunction is still controversial.

Majority of existing SRs focused on rTMS for upper limb motor dysfunction after stroke, only 3 of them reported effects of TBS in subgroups analysis, but there are several problems in these SRs.^[22–24]^ The main problems were as follows: without predefined protocol; incomplete search; absent information and reason of excluded studies; and no discussion on the impact of risk of bias on outcomes (see Appendix 2, http://links.lww.com/MD/D355). None of the 3 SRs did a predefined protocol and searched gray literature. Hsu et al^[23]^and Ling et al^[22]^ suggested TBS may be a useful method for poststroke upper limb motor dysfunction by descriptive analysis due to inadequate data, and did not provide a list of excluded studies. Zhang et al^[24]^ reported that iTBS is more beneficial than cTBS, but they did pooled analysis of all the outcome measurements, so did Ling et al.^[22]^ All the 3 SRs ignored funding sources of included trails. Owing to deficiencies of previous SRs and more relevant trails were carried out,^[18,21,26–28]^ we propose to conduct a SR and meta-analysis according to AMSTAR 2.0 and PRISMA, to assess the effectiveness and safety of TBS for upper limb motor dysfunction in patients with stroke, hoping to provide evidence for clinical practice.

### 3.1 Strengths and limitations

This SR aims to investigate the effectiveness and safety of TBS on poststroke upper limb motor dysfunction and has several strengths. We will include new RCTs in recent years, conduct a comprehensive search, and report in compliance with PRISMA. In addition, we will assess the quality of evidence of each outcome using the GRADE. However, there are still some potential limitations. There may be a language bias since we will only include studies published in English and Chinese, and some relevant trails may be missed. Records will be limited to full-text articles, bias may be introduced.

## Author contributions

**Conceptualization:** Xiao-bo Liu, Jian-guo Zhong, Xi-li Xiao.

**Data curation:** Yu-xi Li, Yi-jie Huang.

**Methodology:** Chi Zhang, Rong-jiang Jin, Tian-yu Liu.

**Resources:** Jian-guo Zhong, Xi-li Xiao.

**Supervision:** Xiao-bo Liu, Yu-xi Li.

**Writing – original draft:** Xiao-bo Liu, Chi Zhang, Yong-guo Liu.

**Writing – review & editing:** Jian-guo Zhong, Xi-li Xiao.

## Supplementary Material

Supplemental Digital Content
